# A Novel Coaxial Transosseous Technique for Ganglion Impar Block in Radiation-Induced Sacrococcygeal Fusion

**DOI:** 10.7759/cureus.89810

**Published:** 2025-08-11

**Authors:** Sarvadarshi Saraswata Mahapatra, Sanjeev Kumar, Vinod Kumar, Seema Mishra, Sachidanand Jee Bharati

**Affiliations:** 1 Department of Onco-Anesthesia and Palliative Medicine, All India Institute of Medical Sciences, New Delhi, New Delhi, IND; 2 Department of Anesthesiology, University of Minnesota School of Medicine, Minnesota, USA

**Keywords:** ganglion impar block, pain management, palliative care, radiation fibrosis syndrome, rectal neoplasms

## Abstract

Perineal pain in patients with pelvic malignancies, such as rectal cancer, can be debilitating and significantly impair quality of life. The ganglion impar block (GIB) is an established interventional technique for managing such pain. However, anatomical changes following pelvic radiotherapy, particularly sacrococcygeal fusion, may render conventional approaches to GIB ineffective or unsafe. We report the case of a 41-year-old male with locally advanced rectal cancer and radiation-induced sacrococcygeal fusion who presented with severe, refractory perineal pain. Pain was poorly controlled despite systemic opioids and adjuvant analgesics, and conventional trans-sacrococcygeal GIB attempts under fluoroscopy failed due to the inability to traverse the fused joint. A novel coaxial transosseous technique was employed. A large-bore (21G) needle was used in a rotatory fashion to drill through the ossified sacrococcygeal joint. Subsequently, a 27G spinal needle was introduced coaxially through the first needle, facilitating precise placement and administration of therapeutic agents under fluoroscopic guidance. The patient reported significant and sustained pain relief. Post-procedure, the patient experienced substantial pain reduction (Numerical Rating Scale 8/10 to 2/10), improved defecation-related symptoms, and enhanced functional status. No procedural complications were observed. Follow-up at one week confirmed ongoing analgesic benefit with reduced opioid requirements. The coaxial transosseous GIB is a safe, effective, and innovative technique for managing refractory perineal pain in patients with radiation-induced sacrococcygeal fusion. It offers a viable alternative when conventional approaches fail and warrants further evaluation through prospective studies.

## Introduction

Perineal pain in patients with locally advanced or recurrent rectal cancer significantly impairs quality of life due to its multifactorial etiology such as tumor infiltration, treatment effects, and complications. Pain is both nociceptive and neuropathic, worsened by tumor progression and treatments such as radiotherapy and chemotherapy. Radiation may cause chronic proctitis, fibrosis, and nerve injury, while agents such as oxaliplatin contribute to peripheral neuropathy. The exacerbation of pain during defecation or ambulation leads to social withdrawal and psychological distress [1]. Though opioids remain standard, interventional approaches such as ganglion impar block (GIB) offer targeted relief by interrupting nociceptive transmission from pelvic organs [2]. GIB, administered through various imaging-guided approaches, has been shown to reduce pain and opioid use significantly. However, post-radiotherapy anatomical changes, such as sacrococcygeal fusion [3], complicate traditional GIB techniques, requiring innovative solutions to achieve effective pain control in affected patients.

## Case presentation

A 41-year-old male with locally advanced rectal cancer and post-chemoradiotherapy status presented to the Palliative Care Unit with a 10-month history of perianal pain that had insidiously progressed to severe aching and burning sensations, rated 8/10 on the Numerical Rating Scale (NRS). The pain radiated to the back and intensified during defecation and ambulation, significantly impairing his social interactions and quality of life.

Despite being on a regimen of oral morphine (10 mg, 1.5 tablets every 4 hours) along with co-analgesics and adjuvants, the patient experienced only partial relief from baseline pain, with acute exacerbations during defecation remaining particularly distressing. Given these concerns, a GIB was planned. However, attempts using the conventional trans-sacrococcygeal approach under C-arm fluoroscopic guidance with a 22G spinal needle were unsuccessful; the needle could not traverse the sacrococcygeal joint due to radiation-induced fusion, leading to needle bending and blunting.

**Figure 1:**
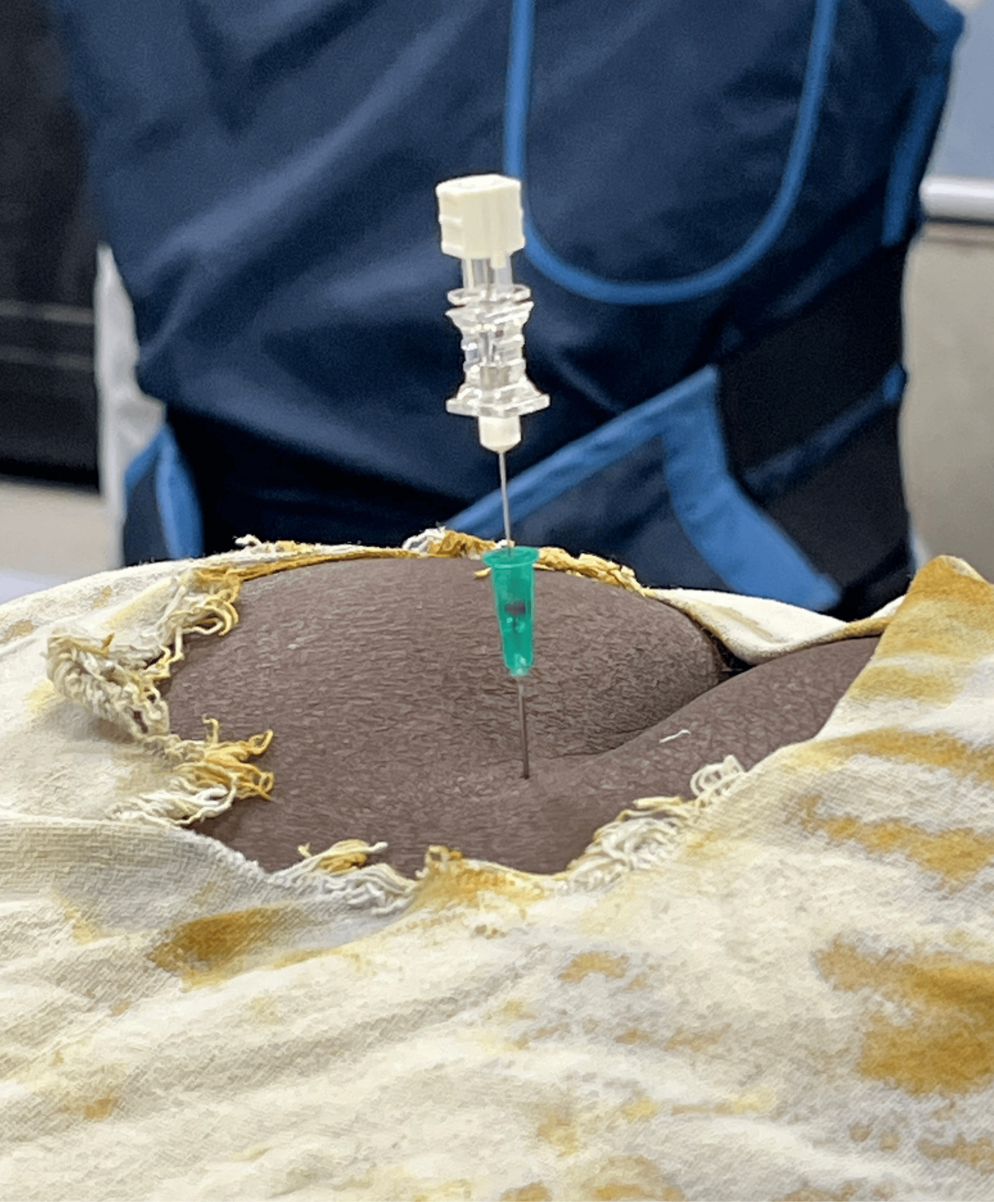
Novel coaxial transosseous technique for ganglion impar block

**Figure 2:**
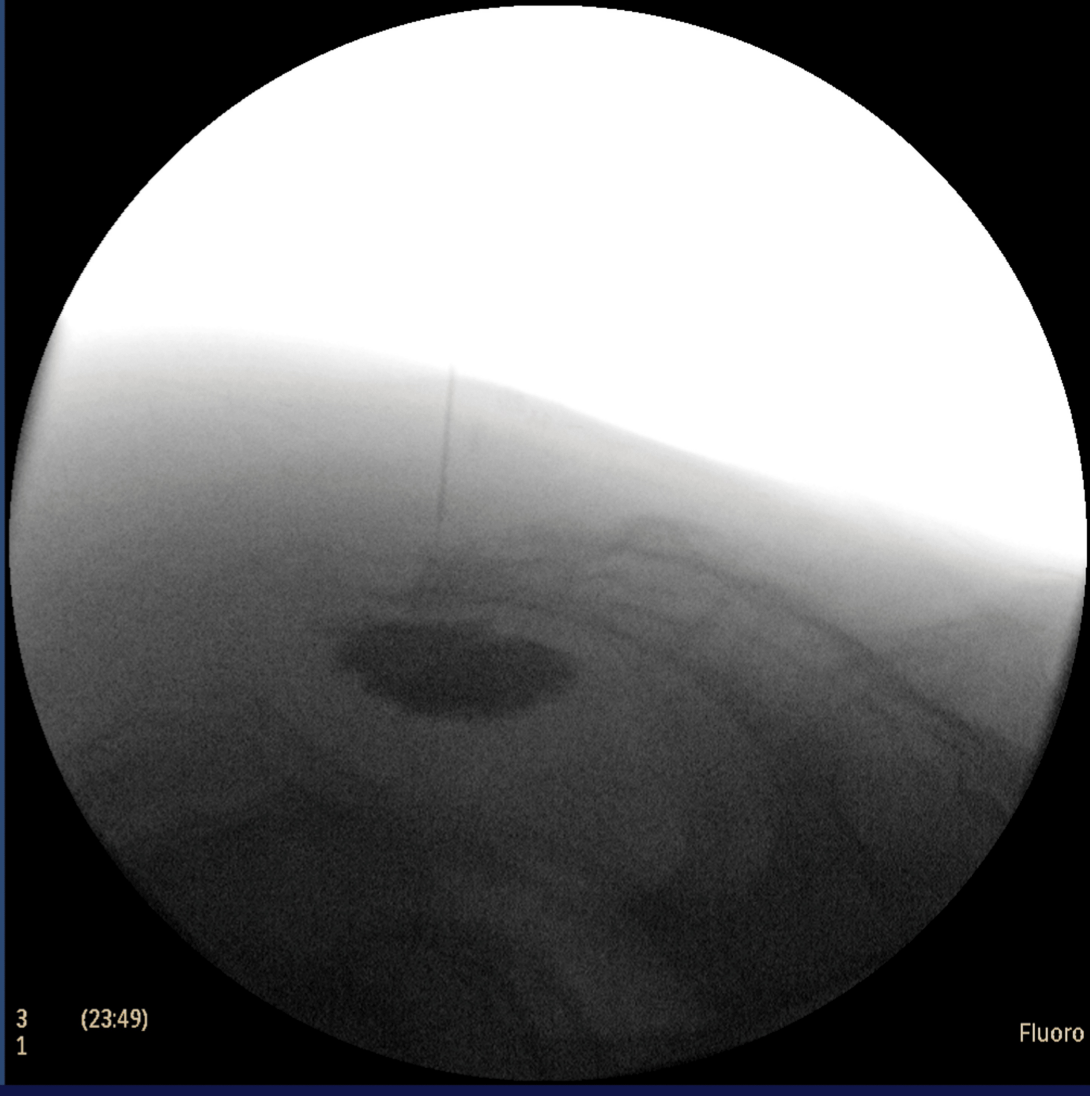
Contrast spread via novel coaxial transosseous technique for ganglion impar block in the patient with post-radiation changes

To overcome this anatomical challenge, we employed a novel coaxial transosseous technique (Figure 1). Initially, a large-bore hypodermic needle (21G, 1.5") was advanced with a rotatory motion to drill through the ossified sacrococcygeal joint. However, attempts to inject the therapeutic agent through this needle were unsuccessful due to clogging by bony debris and the viscosity of the contrast-containing solution. Subsequently, a spinal needle (27G, 3.5") with a stylet was introduced coaxially through the lumen of the larger needle. The stylet prevented clogging, allowing the needle to reach the target location effectively. Upon removal of the stylet, the therapeutic agent was administered seamlessly under fluoroscopic guidance (Figure 2), resulting in significant pain relief (NRS 8 to 2 in 30 minutes). At the one-week follow-up, the patient reported sustained pain relief and a noticeable decrease in opioid use. This outcome aligns with clinical observations that ganglion impar neurolysis can provide significant analgesic benefits and reduce reliance on opioid medications in patients with chronic perineal pain.

This case highlights the utility of the coaxial transosseous approach in administering GIBs for patients with radiation-induced sacrococcygeal fusion, where conventional techniques fail due to anatomical alterations.

## Discussion

Chronic perineal pain, particularly in patients with pelvic malignancies such as rectal cancer, poses significant challenges in pain management. The ganglion impar, a solitary sympathetic ganglion located anterior to the sacrococcygeal junction, serves as a critical relay for nociceptive signals from the perineal region. Blocking this ganglion has been recognized as an effective intervention for alleviating perineal pain [2]. Traditional approaches to GIB, including trans-sacrococcygeal, lateral CT-guided, and blind anococcygeal techniques, have demonstrated efficacy in various clinical scenarios. However, anatomical alterations resulting from treatments such as radiotherapy can complicate these procedures [3].

Radiation-induced sacrococcygeal fusion presents a significant obstacle in performing standard GIB procedures. Normally, the ganglion impar is accessed via the trans-sacrococcygeal or transdiscal approach, where the needle is guided through the sacrococcygeal joint to reach the retroperitoneal space anterior to the coccyx. However, radiotherapy in pelvic malignancies often results in fibrosis, calcification, and even ossification of the sacrococcygeal joint. This fusion eliminates the natural joint space and increases the hardness and density of the tissues, making needle penetration extremely difficult. Attempts to insert a needle through this fused segment often result in needle bending, deflection, or procedural failure. Moreover, these changes obscure anatomical landmarks and may displace or encase the ganglion, complicating localization and increasing the risk of injury to surrounding structures. These factors severely limit the feasibility and safety of conventional GIB techniques in such patients, necessitating alternative or modified approaches for effective pain relief.

In cases in which conventional trans-sacrococcygeal approaches to GIB are impeded by radiation-induced sacrococcygeal fusion, alternative techniques such as the lateral CT-guided and blind anococcygeal approaches have been explored. The lateral CT-guided approach, while valuable in patients with radiation-induced fibrosis or anatomical alterations, presents several challenges [4]. Anatomical variability, especially in patients who have undergone radiotherapy, can make precise needle placement challenging due to variations in the ganglion impar's position. Achieving optimal needle trajectory in this approach can be technically demanding, particularly in the presence of anatomical distortions. Additionally, the proximity of critical structures such as the rectum and blood vessels increases the risk of inadvertent injury. CT-guided procedures may also take longer, potentially leading to patient discomfort and increased procedural costs.

The blind anococcygeal approach, historically used for GIB, involves inserting a needle through the anococcygeal ligament without direct imaging guidance, relying on anatomical landmarks and clinician experience. This technique has notable drawbacks, including the lack of direct visualization, which increases the risk of misplacement and potential injury to adjacent structures and needle stick injury to the practitioner [5]. Individual anatomical differences can further complicate needle placement, making the procedure less predictable. Blind techniques carry a higher risk of adverse events, such as rectal perforation, bleeding, and nerve injury, due to the inability to continuously monitor needle advancement. The absence of real-time guidance can also result in increased patient discomfort during the procedure.

In light of these challenges, we employed a novel coaxial transosseous technique in a 41-year-old male with radiation-induced sacrococcygeal fusion and refractory perineal pain. The novel coaxial transosseous technique described in this case offers a practical and effective solution for administering GIBs in patients with challenging sacrococcygeal anatomy due to radiation-induced fusion. This procedure’s relative simplicity and safety under fluoroscopic guidance make it a valuable addition to the interventional pain management arsenal in oncology and palliative care settings.

## Conclusions

This case underscores the utility of a novel coaxial transosseous approach for GIB in patients with radiation-induced sacrococcygeal fusion, where standard techniques fail due to altered anatomy. By facilitating precise needle placement through ossified tissue using a needle-within-needle strategy, this method ensures effective drug delivery and significant analgesia. The technique is safe, feasible under fluoroscopic guidance, and offers a viable, opioid-sparing intervention for refractory perineal pain in advanced rectal cancer. Its application can be extended to other complex pelvic pain syndromes, making it a valuable tool in palliative and interventional pain management practice.
